# The Bacterial Ecosystem of Mother’s Milk and Infant’s Mouth and Gut

**DOI:** 10.3389/fmicb.2017.01214

**Published:** 2017-06-30

**Authors:** Elena Biagi, Sara Quercia, Arianna Aceti, Isadora Beghetti, Simone Rampelli, Silvia Turroni, Giacomo Faldella, Marco Candela, Patrizia Brigidi, Luigi Corvaglia

**Affiliations:** ^1^Department of Pharmacy and Biotechnology, University of BolognaBologna, Italy; ^2^Neonatal Intensive Care Unit, Department of Medical and Surgical Sciences, University of BolognaBologna, Italy

**Keywords:** infant gut microbiota, infant oral microbiota, milk microbiota, microbiota assembly, breastfeeding, term infants

## Abstract

The progressive building of the infants’ gut microbiota is pivotal for educating their immune system. Human breast milk is among the first sources of microbes for the assembly of the infant’s microbiota, but research struggles to give a demonstration for the origin of bacteria in milk. Aiming at contributing to the knowledge on assembly of the mother’s milk and infant’s microbiome, here we characterized the oral, gut and milk ecosystems in a homogeneous cohort of 36 healthy mother–infants pairs, by 16S rRNA next-generation sequencing. A limited number of operational taxonomic units (OTUs) was shared among the three ecosystems, including not only OTUs assigned to the well-known immune-modulating *Bifidobacterium* genus, but also specific *Streptococcus* and *Staphylococcus* OTUs, which were dominant in the infant’s mouth ecosystem. The high conservation of these OTUs among the three ecosystems seems to call for a worth exploring ecological role through targeted and/or culture-dependent techniques. Notwithstanding the limitations of a 16S rRNA gene-based molecular characterization, we might hypothesize that the baby’s mouth, being the transition point for the milk to reach the intestine, could play a role in both the gut microbiota assembly, via deglutition, and mother’s milk duct colonization, during suction.

## Introduction

The microbiota of individuals with whom a human being has direct and frequent contacts contributes in shaping its microbial communities (Stahringer et al., 2012; Song et al., 2013). This is even more true in the case of breastfed infants and their mothers, where the microbial ecosystems of the latter are the most relevant sources of colonizing microbes for the former (Arrieta et al., 2014). The progressive building of the infants’ microbiota, especially for what concerns the gut ecosystem, is a crucial proceeding for educating their immune system to the delicate balance between tolerance and reactivity that is needed to maintain health throughout the entire human life (Arrieta et al., 2015; Honda and Littman, 2016; Lynch and Pedersen, 2016). Consequently, the understanding of the colonization dynamics of the infant’s gut microbiota is not only fascinating from the ecological point of view, but also incredibly relevant for clinical immunology (Arrieta et al., 2015; Honda and Littman, 2016).

The infant’s gut microbiota is a highly dynamic community that is progressively and continuously shaped during the 1st days of life, with nutrition (breast vs. formula feeding) being among the most relevant drivers for its composition (Gritz and Bhandari, 2015). With its estimated 3 log CFU/ml of bacterial concentration (Jost et al., 2014), human breast milk is listed among the first sources of microbes for the infant’s gut ecosystem, together with the mother’s skin, mouth and vaginal tract, in case of vaginal delivery (Mueller et al., 2015). Research struggles to give a conclusive demonstration for the origin of the bacteria recovered in human milk: even if a controversial “bacterial entero-mammary pathway” has been proposed (Rodríguez, 2014), contamination by the surrounding skin microbiota and other environmental sources might also occur. Indeed, facultative anaerobic or prevalently aerobic species are the major colonizers of the human milk ecosystem: *Streptococcus* and *Staphylococcus* are the most frequently isolated and abundant bacterial groups in milk samples, together with skin-derived or environmental contaminants (i.e., *Propionibacterium* and genera of the Enterobacteriaceae family) (Fitzstevens et al., 2016). However, well-known intestinal probiotic bacteria (i.e., *Bifidobacterium* and *Lactobacillus*) are often retrieved by both molecular and cultivation-based studies (Fitzstevens et al., 2016). Next generation sequencing also allowed the detection of obligate anaerobic, gut-associated genera, such as *Bacteroides*, *Blautia*, *Dorea*, and *Faecalibacterium* (Jost et al., 2013); if alive, these bacteria could act as pioneers in the infant gut for the construction of the adult gut microbiota, which will begin to settle down at weaning (Rodríguez, 2014).

In this scenario of microbial exchange between mother and child, the baby’s mouth is unavoidably involved, being the obligate transition point for the milk to reach the gastrointestinal tract. The oral microbiota is a well-characterized portion of the human microbiome. It is usually dominated by *Streptococcus* and *Staphylococcus* in healthy, breastfed term infants; aerobic or facultative anaerobic bacterial taxa, such as *Gemella*, *Actinomyces*, and *Veillonella*, act as later and minor colonizers (Sampaio-Maia and Monteiro-Silva, 2014). The mouth is a particularly exposed ecosystem, anatomically open to the external environment and continuously in contact with air, food, and water. For these reasons, this ecosystem needs to cope with chemical, physical and mechanical fluctuations. The mouth of healthy individuals is not routinely found to be colonized by non-oral microorganisms, possibly because exogenous bacteria lack in specific adhesins and receptors that would enable them to bind to oral surfaces, or are excluded by immune mechanisms (Wade, 2013). On the contrary, evidences of seeding of the baby’s gut by the oral microbiome have been provided (Costello et al., 2013; Ding and Schloss, 2014).

In this frame, it is crucial to include the bacteria inhabiting the infant’s oral cavity in the complex mechanism of bacterial transfer between the mother’s milk microbiota and the infant’s gut ecosystem. Indeed, the oral ecosystem might contribute in seeding the gut both directly, through deglutition, and indirectly, by contaminating the mother’s milk ducts, during suction.

In an attempt to decipher the relationship between the mother’s milk ecosystem and the infant’s microbiome, we analyzed, to our knowledge for the first time, the microbial composition of oral, gut and milk ecosystems in a small, yet very homogeneous, cohort of 36 healthy mother–infant pairs. By limiting the influence of confounding variables (e.g., delivery mode, gestational age), our findings shed some light on the relevance of bacterial sharing between these ecosystems.

## Materials and Methods

### Subjects and Samples Collection

The Nursery of S. Orsola-Malpighi Hospital in Bologna, Italy, recruited mother–infant pairs meeting the following criteria: (i) vaginal delivery at term (≥37 weeks gestation), (ii) exclusive breastfeeding during the sampling period, (iii) no antibiotic/probiotic exposure of either the mother or the infant during pregnancy, intrapartum or postnatally. Infants who had or developed clinical conditions that required hospitalization were excluded.

Written informed consent was obtained, in accordance with the Declaration of Helsinki, from each mother before the mother–infant pair was discharged from the nursery (48–72 h after delivery). Follow-up visits at 20 days of life were scheduled in order to obtain a neonatal fecal sample, two neonatal oral swabs (before and after breastfeeding), and a fresh mother milk sample. Feces were collected from diapers using a standard sterile collection tube. Milk samples were collected with the aid of a breast pump into sterile plastic tubes; prior to collection, mothers were asked to wash the nipple and mammary areola with soap and water. Oral samples were obtained by gently swabbing a sterile cotton-tipped applicator on the inside of the infant’s cheek. Samples were immediately delivered to the laboratory using cold packs, then split into aliquots ready for DNA extraction and frozen within few hours from collection. Samples were thawed in batches for processing. All samples were processed within 4 months of receipt. Demographic and clinical data were recorded in a specific case report form. The study was approved by the ethics committee of the S. Orsola-Malpighi Hospital in Bologna (study protocol 53/2014/U/Tess). Methods were carried out in accordance with the approved guidelines.

### Total Bacterial DNA Extraction

Total bacterial DNA was extracted from feces using the DNeasy Blood & Tissue Kit (QIAGEN, Hilden, Germany) with a modified protocol, as described by Biagi et al. (2016). Two hundred fifty mg of stools were resuspended in 1 ml of lysis buffer (500 mM NaCl, 50 mM Tris–HCl pH 8, 50 mM EDTA and 4% SDS) and treated with 3 bead-beating steps in a FastPrep instrument (MP Biomedicals, Irvine, CA, United States) at 5.5 movements per sec for 1 min. Successively, samples were heated at 95°C for 15 min. Solid particles were centrifuged at full speed (13000 rpm/19000 rcf) for 5 min at 4°C, then 260 μl of 10 M ammonium acetate were added and the samples incubated for 5 min in ice. Debris were pelleted by 10 min of centrifugation at full speed at 4°C, the supernatants were collected and 1 volume of isopropanol was added. Samples were incubated in ice for 30 min. DNA was collected by 15 min of centrifugation at full speed at 4°C and the pellet washed with 70% ethanol. The pellet was then dissolved in 100 μl of TE buffer and treated with 2 μl of DNase-free RNase (10 mg/ml) for 15 min at 37°C. After incubation, 200 μl of AL buffer (QIAGEN) and 15 μl of proteinase K were added and heated at 70°C for 10 min. DNA was further purified using QIAamp Mini Spin columns (QIAGEN) following the manufacturer’s instructions.

For milk samples, 2 ml of milk were centrifuged at full speed for 10 min at 4°C and then the same protocol described above for fecal samples was applied.

For DNA extraction from oral swabs, the cotton swab was suspended in 500 μl of PBS, vortexed for 1 min and sonicated for 2 min. These 2 steps were repeated twice, then 2 cycles of bead-beating with FastPrep at 5.5 movements per sec for 1 min, with 200 mg of glass beads, were applied. Cotton residues were removed and the debris pelleted by centrifugation at 9000 *g* for 5 min. The supernatant was discarded and the pellet resuspended in 180 μl of enzymatic lysis buffer (QIAGEN). Samples were then treated according to the DNeasy Blood & Tissue kit (QIAGEN) instructions, following the protocol for Gram-positive bacteria.

Extracted DNA was quantified using the NanoDrop ND-1000 spectrophotometer (NanoDrop Technologies, Wilmington, DE, United States).

### 16S rRNA Gene Amplification and Sequencing

For each sample, the V3–V4 region of the 16S rRNA gene was PCR amplified in 25 μl final volume containing 5 μl of microbial DNA (diluted to 5 ng/μl), 2X KAPA HiFi HotStart ReadyMix (KAPA Biosystems, Resnova, Rome, Italy), and 200 nM of S-D-Bact-0341-b-S-17/S-D-Bact-0785-a-A-21 primers carrying Illumina overhang adapter sequences. Thermocycler was programmed as follows: initial denaturation at 95°C for 3 min, 25 cycles of denaturation at 95°C for 30 s, annealing at 55°C for 30 s, and extension at 72°C for 30 s, and a final extension step at 72°C for 5 min (Biagi et al., 2016). Amplicons of about 460 bp were purified with a magnetic bead-based clean-up system (Agencourt AMPure XP; Beckman Coulter, Brea, CA, United States). Indexed libraries were prepared by limited-cycle PCR using Nextera technology and further cleaned up with AMPure XP magnetic beads (Beckman Coulter). Libraries were pooled at equimolar concentrations (4 nM), denatured and diluted to 6 pM before loading onto the MiSeq flow cell. A 2 × 300 bp paired end protocol was used, according to the manufacturer’s instructions (Illumina, San Diego, CA, United States).

### Bioinformatics and Statistics

Raw sequences were processed using a pipeline combining PANDAseq (Masella et al., 2012) and QIIME (Caporaso et al., 2010). Sequencing reads were deposited in the National Center for Biotechnology Information Sequence Read Archive (NCBI SRA, under BioProject ID PRJNA378341). High-quality reads, as selected using the default values in QIIME, were binned into operational taxonomic units (OTUs) according to the taxonomic threshold of 97% using UCLUST (Edgar, 2010), through an open-reference strategy. Taxonomy was assigned using the RDP classifier against Greengenes database (May 2013 release). Chimera filtering was performed by discarding all singleton OTUs. Alpha rarefaction was analyzed by using Chao1 and Shannon index metrics. Beta diversity was estimated by computing weighted and unweighted UniFrac distances.

Statistics was performed using R software^1^ and the libraries vegan and made4. Weighted and unweighted UniFrac distances were used for Principal Coordinates Analyses (PCoA), and the significance of separation was tested by permutational multivariate analysis of variance using the function “adonis” of the vegan package, after testing for homogeneity of dispersion using the function “betadisper.” Wilcoxon test was used to assess significant differences between two groups of samples; adaptations for paired samples were used when necessary. Kruskal–Wallis test was used for multiple comparisons, followed by Tukey *post hoc* test when appropriate. *P*-values were corrected for multiple comparisons using the Benjamini–Hochberg method. *P* < 0.05 was considered as statistically significant. Correlation between datasets was tested by using the Kendall method.

## Results and Discussion

Thirty-six mother–infant pairs were included in the study. All infants were vaginally delivered and exclusively breastfed. Neither infants nor mothers had received any antibiotics or probiotics until the sampling date. One-hundred-forty-three samples were collected 20 days after delivery: 36 mother’s milk samples, 36 infant’s fecal samples, and 71 infants’ oral swabs (35 pairs of pre and post-breastfeeding samples plus one unpaired pre-breastfeeding sample).

The extracted bacterial DNA was phylogenetically characterized by 16S rRNA gene (V3–V4 region) Illumina sequencing. A total of 1,475,619 high-quality reads was obtained with a mean of 10,319 ± 3,364 reads per sample. Rarefaction curves obtained with Shannon and Chao1 metrics approximated the saturation level after 3,000 reads (Supplementary Figure S1). Reads were clustered in 7,524 operational taxonomic units (OTUs) at 97% of identity. OTU table and taxa summary tables at family and genus level are available as **Supplementary Table S1**. Family level histograms for each sample are reported in Supplementary Figures S2–S5.

**FIGURE 1:**
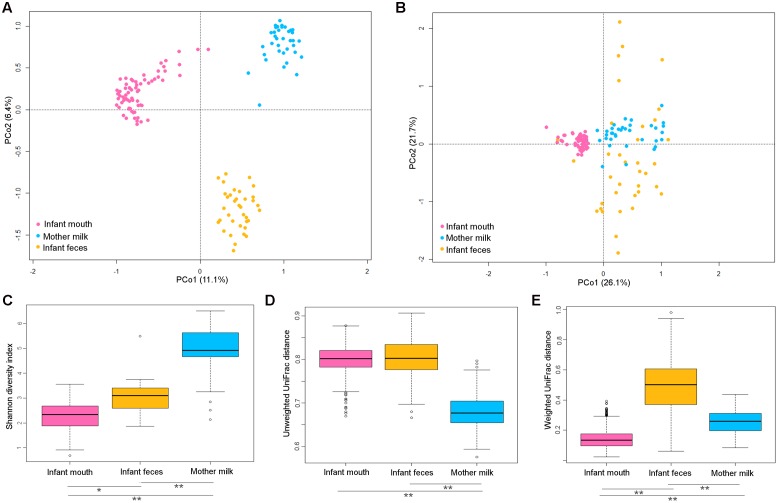
Diversity in the bacterial ecosystem of the mother’s milk, and infant’s feces and mouth. PCoA based on unweighted **(A)** and weighted **(B)** UniFrac distances of the microbiota of mother’s milk (light blue), infant feces (yellow), and infants mouth (pink). Samples are identified by filled circles. In both PCoA first and second principal components (PCo1 and PCo2) are plotted. The percentage of variance in the dataset explained by each axis is reported. Box and whiskers distribution of the Shannon α-diversity index **(C)**, intra-group unweighted UniFrac distances **(D)**, and intra-group weighted UniFrac distances **(E)**, calculated for milk (light blue), fecal (yellow) and oral (pink) samples. Significant differences between datasets are indicated, as calculated using Tukey *post hoc* test after Kruskal–Wallis test for multiple comparisons (^∗^*P* ≤ 0.001, ^∗∗^*P* ≤ 0.0001).

A PCoA based on unweighted UniFrac distance showed that the microbiota of infants’ oral swabs, infants’ feces and mothers’ milk clustered separately (**Figure 1A**), as expected being the resident communities of three distinct body districts that are different for pH, oxygen levels, and nutrients availability. Adonis test confirmed that the reported separation was significant, even if this result needs to be taken into account cautiously since the test for homogeneity of dispersion (function betadisper) returned that milk samples had a significantly different dispersion compared to the other groups of samples. When weighted UniFrac distances were used for PCoA (**Figure 1B**), fecal samples overlapped with milk samples on PCo1. Thus, the difference between milk and fecal ecosystems was better explained by unweighted metrics, hinting that it might reside in fractions of the microbial communities that are exclusive of one of the two ecosystems (Lozupone et al., 2007). Fecal samples also showed higher dispersion, indicating higher variability in the most abundant species of the ecosystem, with respect to oral and milk communities.

**FIGURE 2:**
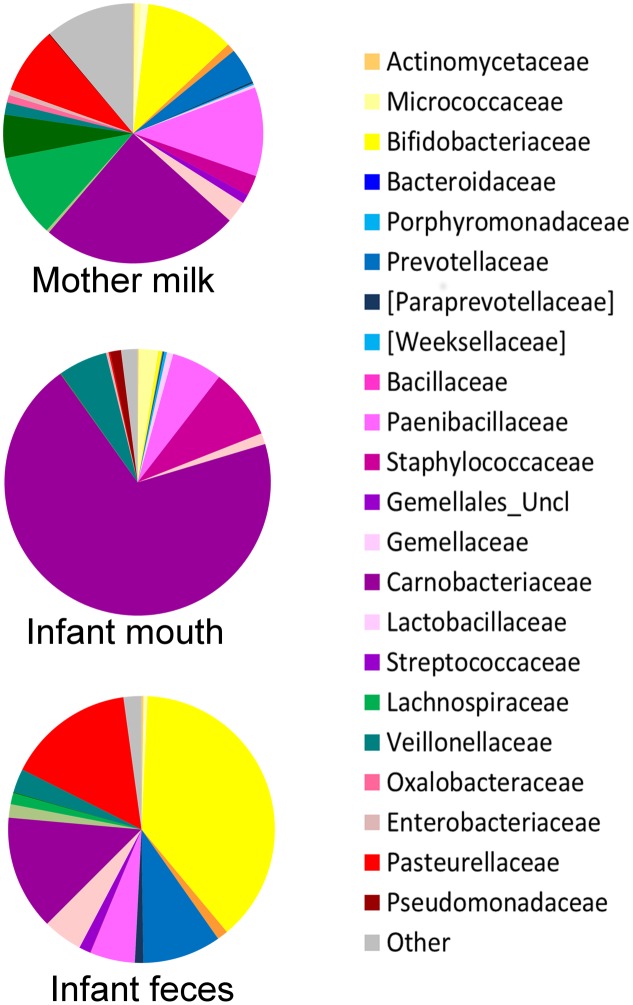
Average composition of the bacterial community in mother’s milk and infant’s mouth and feces. For each group of samples, a pie chart based on the average relative abundance (%) at family level is plotted. Bacterial families with relative abundance ≥0.2% in at least 10% of the samples are depicted. Colors for each family are reported in the legend.

The oral microbiome was the least diverse of the considered ecosystems (Shannon diversity index, mean ± standard deviation (SD), 2.3 ± 0.6; **Figure 1C**), largely dominated by Streptococcaceae (average relative abundance (rel. ab.), 69.8%) (**Figure 2**), with *Streptococcus* being the dominant genus in 94% of samples, confirming the known literature on this ecosystem (Zaura et al., 2014; Lif Holgerson et al., 2015; Hendricks-Muñoz et al., 2015; Davé et al., 2016). Also confirming the large amount of knowledge on the topic (Jost et al., 2012; Mueller et al., 2015), the fecal microbiota of breastfed infants at 20 days of life was dominated by Bifidobacteriaceae (average rel. ab., 38.2%), with *Bifidobacterium* being the dominant genus in 67% of samples. Fecal microbiota also included relevant average abundances of Enterobacteriaceae (15.4%), Streptococcaceae (13.9%), Bacteroidaceae (9.5%), Staphylococcaceae (5.4%), and Lactobacillaceae (4.8%) (**Figure 2**).

Both oral and fecal microbiota of infants showed higher unweighted UniFrac distances within group (0.80 ± 0.03 and 0.80 ± 0.04, respectively) compared to milk (0.68 ± 0.04, Kruskal–Wallis test, *P* < 0.0001). When the weighted UniFrac metric was considered, the within-group distances obtained for the fecal samples remained the highest (0.5 ± 0.2), whereas those calculated for the oral samples became the lowest (0.14 ± 0.06 Kruskal–Wallis test, *P* < 0.0001). Since weighted UniFrac measure is better suited to detect differences in abundance even when the overall groups of organisms that are present in each sample remain the same (Lozupone et al., 2007), these observations suggest that the variability between oral samples might reside in subdominant species that are not highly conserved among samples.

The average microbiota profile obtained for breast milk was significantly more diverse (Shannon diversity index = 4.9 ± 1.1; **Figure 1C**) than both infant’s feces and oral swabs (3.0 ± 0.7 and 2.3 ± 0.6, respectively; Kruskal–Wallis test, *P* < 0.0001); interestingly, according to the unweighted UniFrac metric, the variability among milk samples was the lowest (0.68 ± 0.03; Kruskal–Wallis test *P* < 0.0001; **Figure 1D**), and remained significantly lower than that of fecal samples when the weighted UniFrac distances were computed (0.25 ± 0.07; *P* < 0.0001; **Figure 1E**). In other words, the milk ecosystem of the 36 enrolled mothers was richer and more similarly composed among samples (in terms of bacterial species) than the fecal or mouth ecosystem of their children, suggesting that the milk duct might act as an environmental filter allowing for the survival and proliferation of the same bacterial species in most individuals (a “niche-based” community assembly, according to Costello et al. (2012). As expected (Fitzstevens et al., 2016), the milk ecosystem phylogenetic structure showed a slight dominance of Streptococcaceae (average rel. ab., 24.5%), with *Streptococcus* being the dominant genus in 53% of samples, but also a considerable representation of the typically infant fecal family Bifidobacteriaceae (11.2%, with *Bifidobacterium* being the dominant genus in 19% of samples) and Staphylococcaceae, which is instead a common skin and mouth inhabitant (Belkaid and Segre, 2014) (11.1%, with *Staphylococcus* being the dominant genus in 11% of samples) (**Figure 2**). Confirming previous studies (Jost et al., 2013, 2014), and supporting the hypothesis of a possible link between the milk microbiota and the gut ecosystem of the mother, also anaerobic bacterial families that are commonly found in the adult human intestine, such as Lachnospiraceae, Ruminococcaceae, and Bacteroidaceae, were present with an average rel. ab. of 10.3, 5.4, and 4.4%, respectively. Specifically designed studies are required to investigate if these bacteria are indeed alive in the milk ecosystem, as well as the ecological importance of this putative bacterial migration from the mother’s gut to the milk duct.

The milk microbiota was the only one showing a few genera that were present in more than half of the subjects but never retrieved in the other two ecosystems, partly confirming the observations of Jost et al. (2014). In particular, sequences assigned to the genus *Ralstonia* were detected at a relative abundance >0.1% in 83% of the milk samples (average rel. ab., 0.67%), and in none of the infant’s fecal or oral samples; similar trends were shown by the genus *Sediminibacterium* (detected in 61% of the milk samples, average rel. ab., 0.15%) and unclassified members of the Flavobacteriaceae family (detected in 50% of the milk samples, average rel. ab., 0.16%).

No correlation between the relative abundance of each family or genus in the three different ecosystem was found, after *P*-values correction, with the exception of the abundance of the subdominant family Lactobacillaceae, whose values were positively correlated in saliva and feces of the same infant (Kendall tau = 0.61, *P* = 0.05).

**FIGURE 3:**
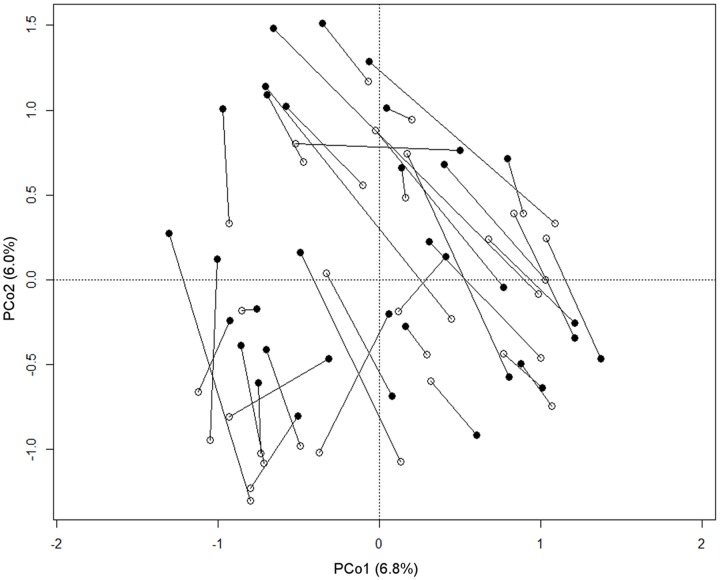
Relationship between pre- and post-breastfeeding infant oral microbiota. PCoA based on unweighted UniFrac distances of the microbiota of the infant’s mouth sampled before (empty circles) and after (filled circles) breastfeeding. Samples from the same subject are connected by a black line. The first and second principal components (PCo1 and PCo2) are plotted. The percentage of variance in the dataset explained by each axis is reported.

According to our observations, the passage of the milk through the mouth affects the composition of the oral microbiota in each infant. Indeed, samples from the same subjects were rarely plotted closer to each other than to samples taken from other babies on the PCoA based on unweighted UniFrac distance (**Figure 3**), even if the multivariate analysis showed no significant separation between the two groups of samples (before and after breastfeeding). Significant differences were not found comparing the genus-level profiles of the samples before and after breastfeeding. However, it was possible to notice that 77% of the enrolled babies showed higher coordinate values on the PCo2 axis in the post-breastfeeding sample than in the pre-breastfeeding one. PCo2 was found to account for more variation in data than expected by random chance (broken-stick eigenvalue = 1.07, actual eigenvalue = 1.17). Indeed, the difference between pre- and post-breastfeeding PCo2 coordinates was found significant (paired Wilcoxon test, *P* = 0.02), meaning that it could be possible to find a common trend in the small changes occurring in the infant’s mouth ecosystem after the mother’s milk passage, in the frame of the individual microbiota structure. Pursuing the identification of these small changes, we found that the coordinate values on the PCo2 axis of each samples were significantly (*P* < 0.01) and positively correlated to the relative abundance of a few bacterial families, which were found averagely more represented in the breast milk than in the infant’s oral ecosystem, such as Lachnospiraceae (Kendall tau, 0.49; average rel. ab.: 0.07% in infant’s mouth, 1.3% in infant’s feces, and 10.3% in mother’s milk), Ruminococcaceae (Kendall tau, 0.46; average rel. ab.: 0.04% in infant’s mouth, 0.1% in infant’s feces, and 5.4% in mother’s milk), Oxalobacteriaceae (Kendall tau, 0.40; average rel. ab.: 0.29% in infant’s mouth, 0% in infant’s feces, and 0.72% in mother’s milk), and Bacteroidaceae (Kendall tau, 0.48; average rel. ab.: 0.09% in infant’s mouth, 9.5% in infant’s feces, and 4.4% in mother’s milk).

**FIGURE 4:**
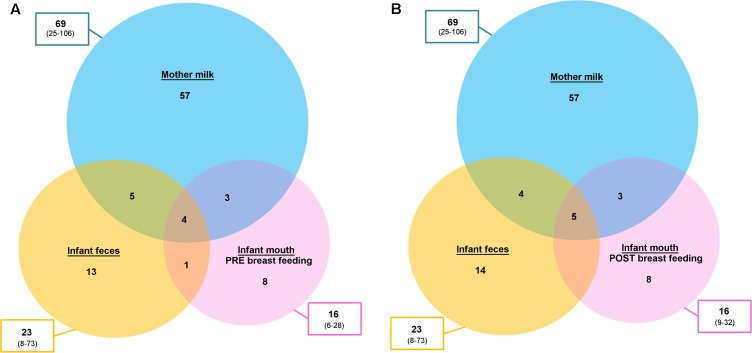
Operational taxonomic unit (OTU) sharing between mother’s milk, and infant’s fecal and oral bacterial ecosystems. Venn diagrams showing the average number of OTUs shared between the bacterial communities of the mother’s milk (light blue), the infant’s feces (yellow) and the infant’s mouth (pink), the latter sampled before **(A)** and after **(B)** breastfeeding. The total number of OTUs for each ecosystem is reported in the boxes outside the circles, expressed as mean and range (in brackets).

Aiming at exploring the possibility of passage of bacteria from one ecosystem to another, we focused our attention to the OTUs shared between two or three samples taken from the same mother–infant pair. It is important to remember that 16S rRNA gene-based characterization does not allow for strain-level analysis. However, the sharing of the same OTUs might give indications on what species could be interesting to further explore (and possibly identify to the strain level) using culture-based techniques and/or metagenomics. Filtering for the OTUs accounting for at least 0.1% of the ecosystem diversity (number of normalized sequences per sample), a mean of 4.5 (considering pre-breastfeeding oral samples, range 2–10) and 4.7 (considering post-breastfeeding oral samples, range 1–11) OTUs were shared between the three ecosystems (**Figure 4**). Among those more frequently shared (**Table 1**) we found OTUs assigned to *Staphylococcus* spp. [shared by the three samples in 80% (pre-breastfeeding) and 83% (post-breastfeeding) of pairs, and by feces and milk only in 91% of cases], *Streptococcus* spp. [shared by the three samples in 80% (pre-breastfeeding) and 83% (post-breastfeeding) of pairs, and by feces and oral samples only in 86% (pre-breastfeeding) and 91% (post-breastfeeding) of cases], and *Streptococcus infantis* (shared by the three samples in 63% of pairs, and by feces and milk only in 97% of cases). Interestingly, the *Streptococcus* OTUs found to be preserved among two or three ecosystems were also the dominant ones in all the infant’s oral samples: indeed, one or a couple of these OTUs generally accounted for the totality of the Streptococcaceae population and, in most cases, for the dominant portion of the entire ecosystem, confirming previous findings on the adult’s oral microbiota (Li et al., 2013). Even if the genera *Streptococcus* and *Staphylococcus* have been recognized as universally predominant in the human milk by a recent systematic review (Fitzstevens et al., 2016), the mechanisms of their colonization of the milk ducts are not explained. The very high abundance of *Streptococcus* in the baby’s mouth that we report in the present study, together with the identity between the dominant *Streptococcus* OTUs in the infant’s mouth and those detected in their mothers’ milk, bring us to suggest that the infant’s mouth could have a seeding effect on the milk duct resident community during suction.

**Table 1:** Operational taxonomic unit (OTU) sharing among the bacterial ecosystem of the infant’s mouth and feces, and the mother’s milk.

	Mother–infant pairs (%) in which the same OTU was present in ≥2 ecosystems					
OTU ID	M-Opre-F	M-Opost-F	M-Opre	M-Opost	M–F	Assigned taxonomy (within Bacteria kingdom)
24152	23	20	31	20	46	p_Actinobacteria; c_Actinobacteria; o_Bifidobacteriales; f_Bifidobacteriaceae; g_Bifidobacterium; s_breve
24615	63	63	97	97	63	p_Firmicutes; c_Bacilli; o_Lactobacillales; f_Streptococcaceae; g_Streptococcus; s_infantis
36228	3	3	51	51	3	p_Firmicutes; c_Bacilli; o_Gemellales; f_Gemellaceae; g_; s_
36370	23	37	34	60	60	p_Proteobacteria; c_Gammaproteobacteria; o_Enterobacteriales; f_Enterobacteriaceae; g_; s_
41720	26	29	29	34	74	p_Actinobacteria; c_Actinobacteria; o_Bifidobacteriales; f_Bifidobacteriaceae; g_Bifidobacterium; s_longum
90869	0	0	3	0	51	p_Actinobacteria; c_Actinobacteria; o_Bifidobacteriales; f_Bifidobacteriaceae; g_Bifidobacterium; s_bifidum
94290	80	83	86	91	83	p_Firmicutes; c_Bacilli; o_Lactobacillales; f_Streptococcaceae; g_Streptococcus; s_
99920	80	83	80	83	91	p_Firmicutes; c_Bacilli; o_Bacillales; f_Staphylococcaceae; g_Staphylococcus; s_
101886	37	37	43	43	43	p_Firmicutes; c_Bacilli; o_Lactobacillales; f_Streptococcaceae; g_Streptococcus; s_

*M, mother’s milk ecosystem; Opre, infant’s oral ecosystem before breastfeeding; Opost, infant’s oral ecosystem after breastfeeding; F, infant’s fecal ecosystem. The OTUs most frequently (>40% of the mother–infant pairs) shared by at least two bacterial ecosystems are shown, with assigned taxonomy. Only OTUs present at a relative abundance >0.1% were considered. Assigned taxonomy is reported using Greengenes syntax*.

Milk and infant’s oral microbiota also shared the presence of a OTU assigned to unclassified members of Gemellaceae family, in 51% of mother–infant pairs; indeed, *Gemella* is another known major core genus in both adult and infant’s oral mucosa (Costello et al., 2013; Zaura et al., 2014; Hendricks-Muñoz et al., 2015).

Most interestingly, the majority of the OTUs shared between mother’s milk and infant’s feces, but not present in infant’s mouth, was assigned to members of the *Bifidobacterium* genus, well-known inhabitants of the gut microbiota of breastfed infants (Arrieta et al., 2014) In particular, OTUs assigned to *Bifidobacterium breve*, *B. bifidum*, and *B. longum* were shared by 46, 51, and 74% of the milk and fecal samples taken from the same mother–infant pair (**Table 1**), supporting the hypothesis that the mother’s milk may act as a reservoir of pioneer probiotic bacteria for the baby’s gut microbiome (Jost et al., 2013). These bacteria are necessary for the degradation of human milk oligosaccharides (HMO) and are boosted in the infant’s gut by the continuous refueling of these energy source (Mueller et al., 2015). It was not surprising to find that bifidobacteria were almost absent in the infant’s oral ecosystem (average rel. ab., 0.4%), probably due to the aerobic environment provided by the baby’s mouth; however, thanks to their known ability to tolerate oxygen exposure (Bottacini et al., 2014), they could be able to survive the transition through the oral cavity without actively colonizing it.

Our study has the limitations of a 16S-based molecular characterization, namely the possible biases deriving from the DNA extraction method, PCR amplification, and OTU assignment algorithm (Schloss and Westcott, 2011; Walker et al., 2015), as well as the failure in discriminating between DNA deriving from live and actively proliferating bacteria and DNA fragments from dead cells. However, the homogeneity of our cohort, as well as the inclusion of oral samples from the infants before and after breastfeeding, led to interesting and useful observations that add knowledge to the complex, and still to be disentangled, topic of the microbiome assembly.

In particular, we observed a very limited number of shared OTUs and reported no correlation between the abundances of bacterial families or genera among the mother’s milk, the child’s mouth and the child’s gut. These findings seem to support the hypothesis that, for most of the inhabiting species, the process of microbiota assembly in different infant’s body sites and in the mothers’ milk ducts is driven more by local adaptation than by true immigration of bacteria from other ecosystems, according to the metacommunity theory depicted by Costello et al. (2012).

On the contrary, an interesting behavior was observed for OTUs assigned to the genera *Bifidobacterium*, *Streptococcus*, and *Staphylococcus*, which constitute a relevant fraction of the infant gut and mouth ecosystem. Indeed, among the OTUs assigned to these genera, a few were retrieved as dominant or very abundant in the majority of the infants and were also shared by the corresponding mother’s milk. Even if the sharing of the same OTUs cannot be considered as a proof of transmission, the colonization of both the mother’s milk and infant’s feces by the same *Bifidobacterium* OTUs seems to sustain the hypothesis that the human milk is among the sources for the baby’s gut inoculation of this bacterial group. At the same time, it does not constitute a proof that live bacteria can be translocated through an entero-mammary pathway (Rodríguez, 2014). Indeed, more recent observations (Meadow et al., 2015) seem to imply that bacteria do not need to be transported through complex enteric mechanisms to migrate from one human ecosystem to other body sites or, possibly, to other individuals, but just to be “emitted” in the microbial cloud that surrounds each individual.

The very frequent retrieval of the same *Streptococcus* and *Staphylococcus* OTUs in the majority of the infants, as well as in their mothers’ milk microbiota, is also an intriguing observation, because this consistency of behavior among the enrolled subjects might call for the existence of a biological or ecological role for these bacteria during the infant’s microbiota assembly. A streptococcal and/or staphylococcal migration from one ecosystem to another cannot be proven by our results, but the very high abundance of *Streptococcus* spp. in the oral ecosystem leads us to speculate that the baby’s mouth might be the among the sources of contamination of both the infant’s gut ecosystem, via deglutition, and mother’s milk ducts, during suction. This will need to be proven by cultivation-based studies, where strains can be isolated and fully characterized, as well as by studies with a longitudinal layout for all the considered ecosystems. Moreover, since all the enrolled infant were born in the same hospital, it cannot be excluded that the frequent and abundant retrieval of the same *Streptococcus* and *Staphylococcus* OTUs might be linked to the contact with the same environment in the very first days of life; this observation could yet strengthen the importance of the living environment in determining the human microbiome composition.

## Conclusion

The assembly dynamics of the infant’s gut ecosystem are a topic of huge interest for human immunology and microbiology (Lynch and Pedersen, 2016). Indeed, the existence of a crucial window of time in which the microbiota contributes to the education of the infant’s immune system has been demonstrated (Arrieta et al., 2015; Honda and Littman, 2016). Our study highlights that bacterial communities in other body sites could be involved in the early phases of the gut microbiota assembly. Even if the specific conditions (pH, oxygen level, nutrient availability) of the infant’s gut seem to be the most relevant filter impacting on its final phylogenetic structure and abundance profile, other bacteria-colonized districts and/or the bacteria-coated body surfaces of the mother might act as a reservoir of seeding species, among which those ecologically necessary and intestinally adaptable will be selected.

## Author Contributions

Conceptualization: PB, GF, and LC. Ethical Committee Application: AA. Experimental investigation: SQ, EB, and IB. Bioinformatics and statistics: EB, SQ, and SR. Writing – Original Draft: EB and SQ. Writing – Review and editing: EB, MC, AA and ST. Samples collection and data curation: AA, IB, and SQ. Figures preparation: EB. Funding acquisition: PB, MC, and EB. All authors discussed the results and commented on the manuscript.

## Conflict of Interest Statement

The authors declare that the research was conducted in the absence of any commercial or financial relationships that could be construed as a potential conflict of interest. The reviewer TA and handling Editor declared their shared affiliation, and the handling Editor states that the process nevertheless met the standards of a fair and objective review.

## Footnotes

1 https://www.r-project.org/

## Supplementary Material

TABLE S1 OTU table, family level relative abundance profiles, and genus-level relative abundance profiles of all samples sequenced in the study.
