# Supplementary material for: The Bacterial Ecosystem of Mother’s Milk and Infant’s Mouth and Gut
Source: Front Microbiol. 2017 Jun 30;8:1214. doi: 10.3389/fmicb.2017.01214 (PMC5491547; doi:10.3389/fmicb.2017.01214)

**Supplementary Figure S1.** Rarefaction curves of each oral (A, B), fecal (C, D), and breast milk (E, F) samples, obtained using Shannon (A, C, E) and Chao1 (B, D, F) metrics for α-diversity measurement.

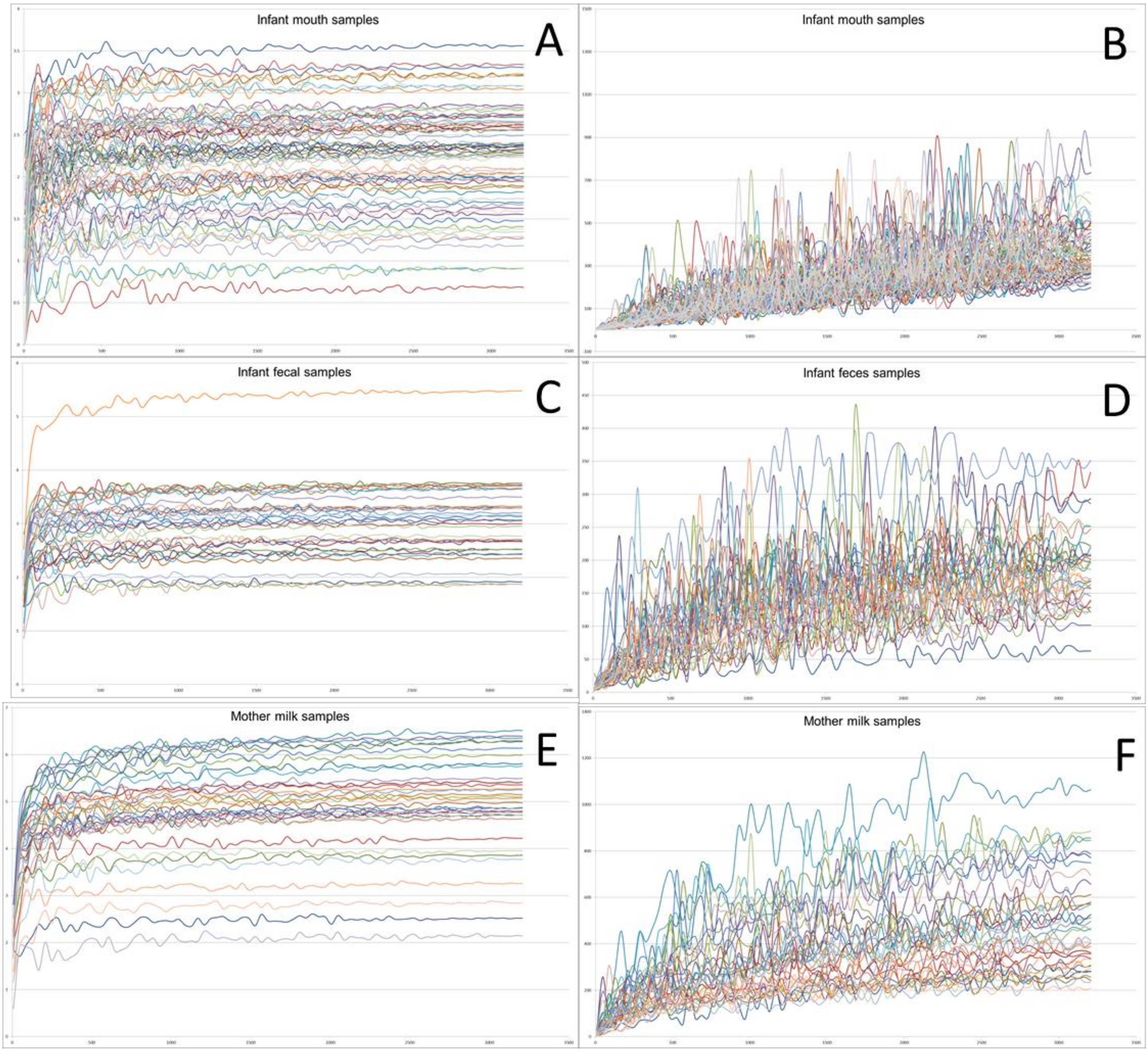

**Supplementary Figure S2.** Family-level phylogenetic profiles of all infants' fecal samples. Bacterial families with relative abundance ≥0.2% in at least 10% of the samples are depicted.

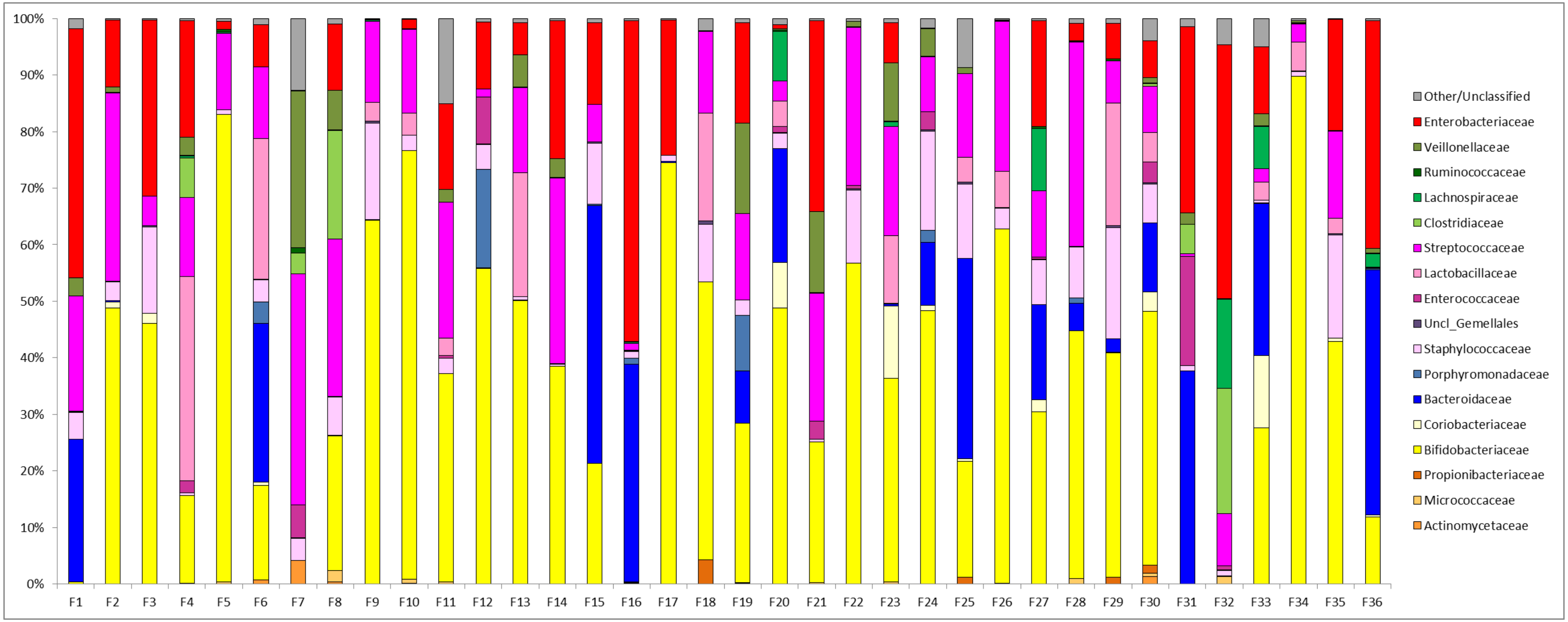

**Supplementary Figure S3.** Family-level phylogenetic profiles of all breastmilk samples. Bacterial families with relative abundance ≥0.2% in at least 10% of the samples are depicted.

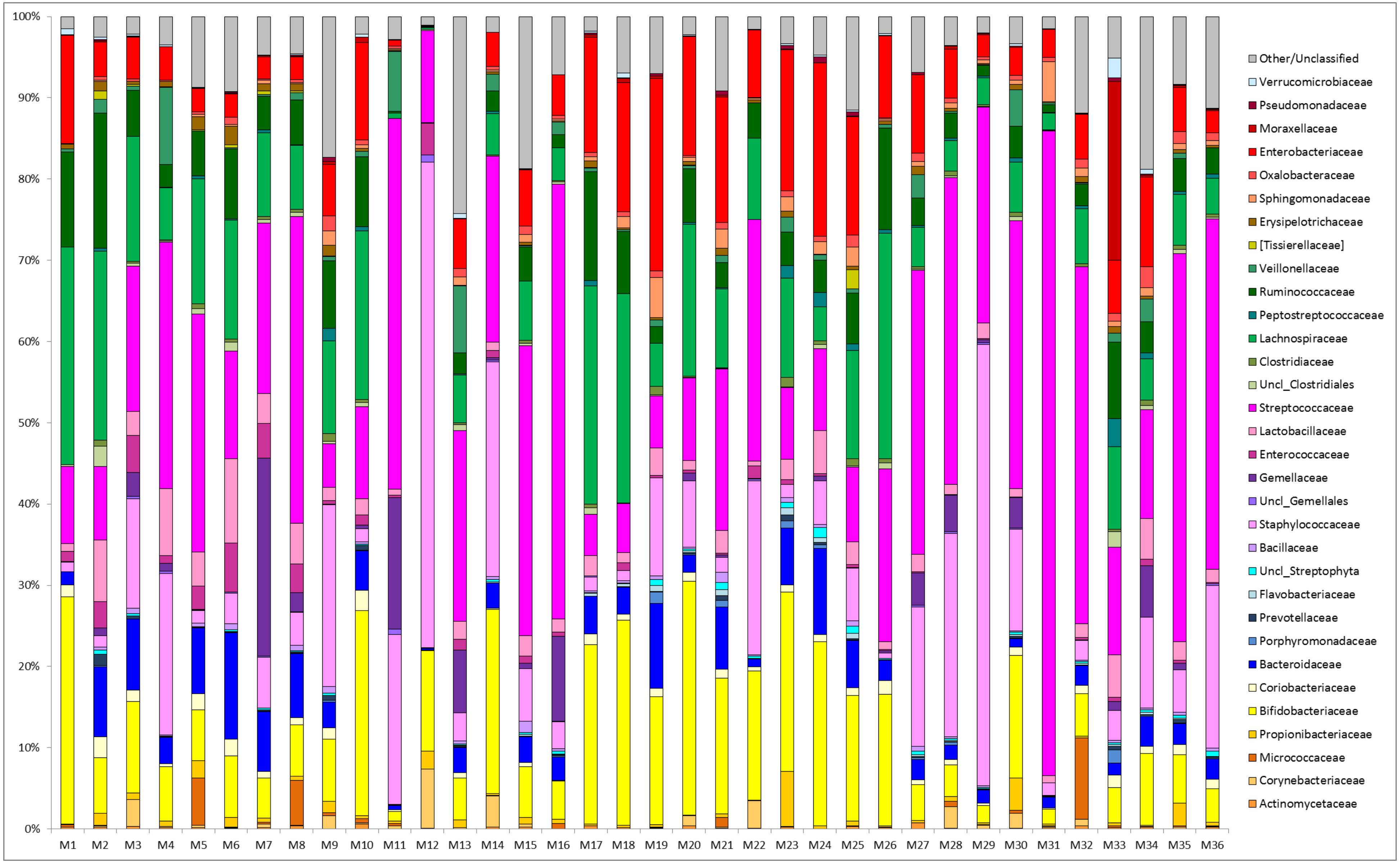

**Supplementary Figure S4.** Family-level phylogenetic profiles of all infants' oral samples taken before breastfeeding. Bacterial families with relative abundance ≥0.2% in at least 10% of the samples are depicted.

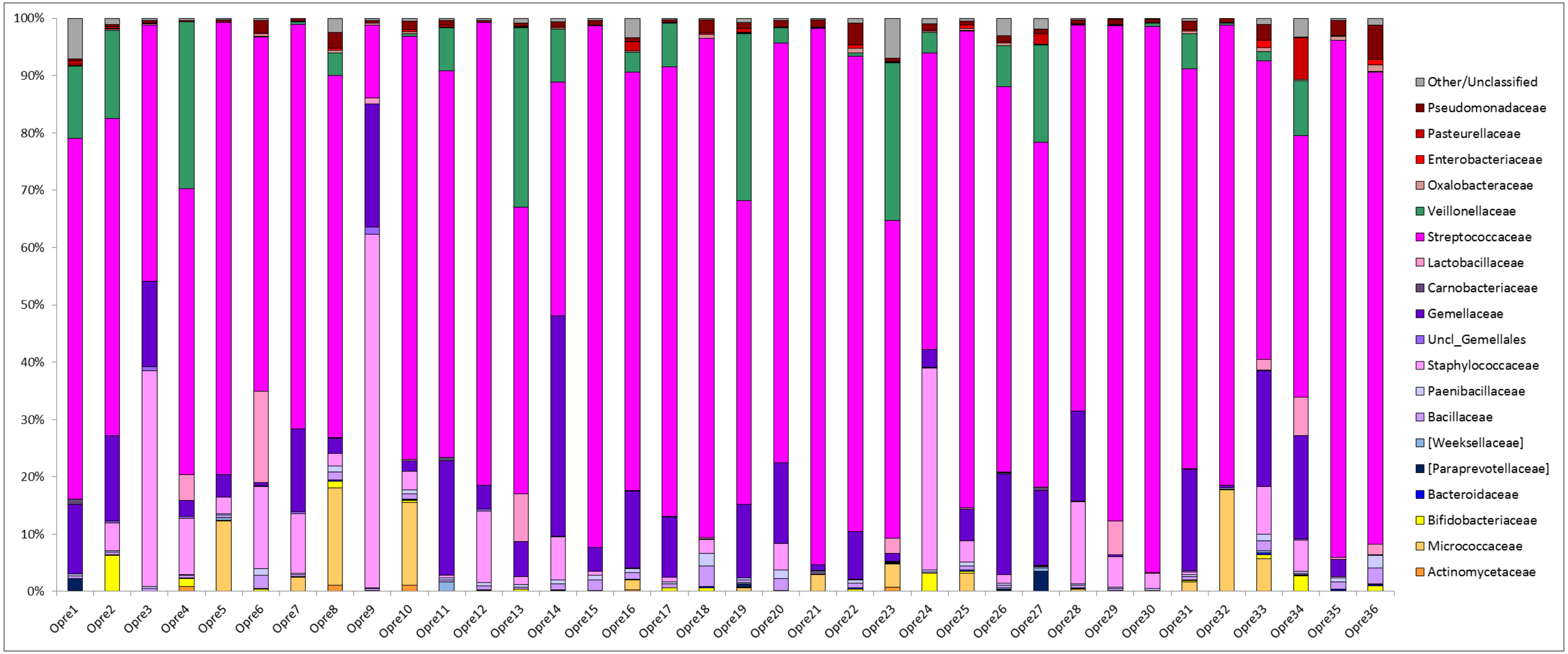

**Supplementary Figure S5.** Family-level phylogenetic profiles of all infants' oral samples taken after breastfeeding. Bacterial families with relative abundance ≥0.2% in at least 10% of the samples are depicted.

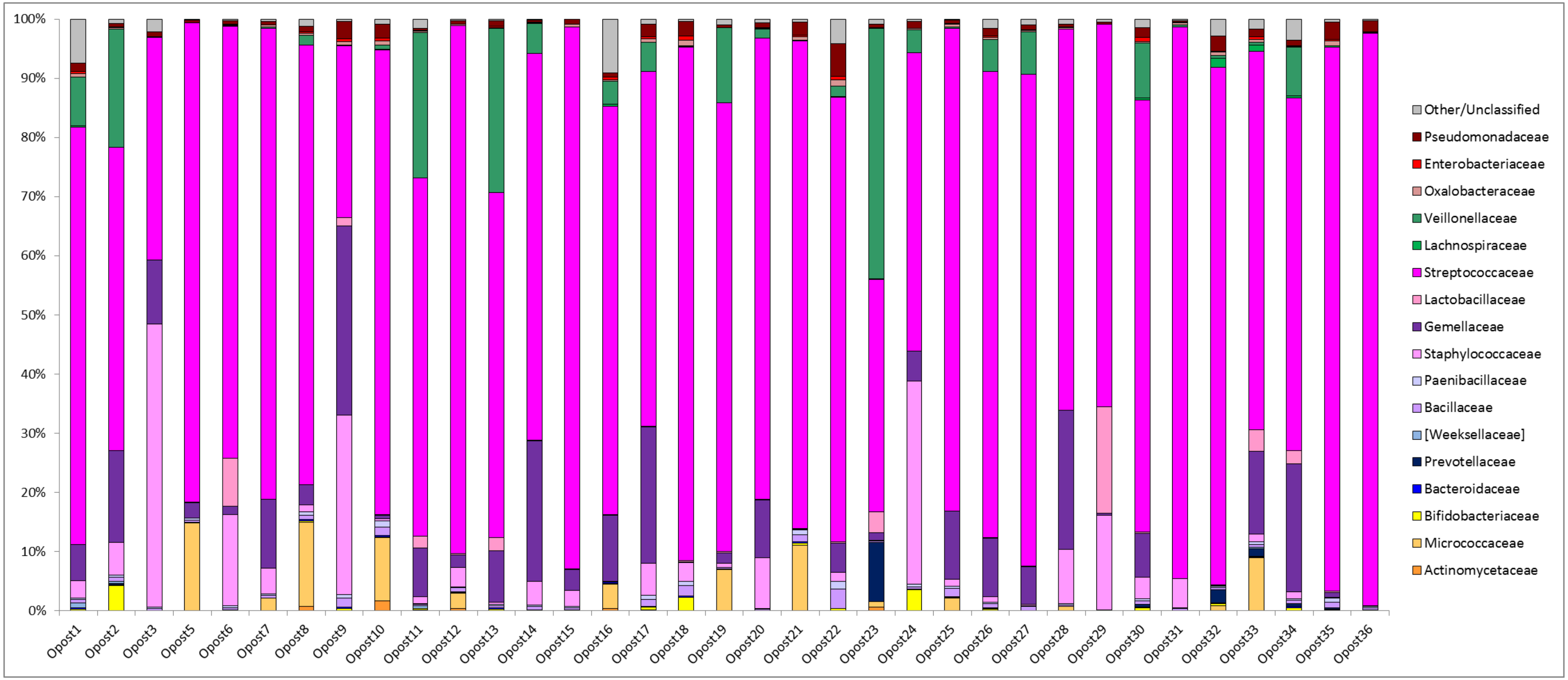

Supplement: Supplementary file 2 [file Image_1.PDF]
